# PPARG: A Promising Therapeutic Target in Breast Cancer and Regulation by Natural Drugs

**DOI:** 10.1155/2023/4481354

**Published:** 2023-06-08

**Authors:** De-Hui Li, Xu-Kuo Liu, Xiao-Tong Tian, Fei Liu, Xu-Jiong Yao, Jing-Fei Dong

**Affiliations:** ^1^The First Affiliated Hospital of Hebei University of Chinese Medicine, Hebei Province Hospital of Chinese Medicine, Shijiazhuang 050011, China; ^2^Graduate School of Hebei University of Chinese Medicine, Shijiazhuang 050091, China; ^3^Hebei University of Chinese Medicine, Shijiazhuang 050091, China

## Abstract

Breast cancer (BC) is the most common type of cancer among females. Peroxisome proliferator-activated receptor gamma (PPARG) can regulate the production of adipocyte-related genes and has anti-inflammatory and anti-tumor effects. Our aim was to investigate PPARG expression, its possible prognostic value, and its effect on immune cell infiltration in BC, and explore the regulatory effects of natural drugs on PPARG to find new ways to treat BC. Using different bioinformatics tools, we extracted and comprehensively analyzed the data from the Cancer Genome Atlas, Genotype-Tissue Expression, and BenCaoZuJian databases to study the potential anti-BC mechanism of PPARG and potential natural drugs targeting it. First, we found that PPARG was downregulated in BC and its expression level correlates with pathological tumor stage (pT-stage) and pathological tumor-node-metastasis stage (pTNM-stage) in BC. PPARG expression was higher in estrogen receptor-positive (ER+) BC than in estrogen receptor-negative (ER−) BC, which tends to indicate a better prognosis. Meanwhile, PPARG exhibited a significant positive correlation with the infiltration of immune cells and correlated with better cumulative survival in BC patients. In addition, PPARG levels were shown to be positively associated with the expression of immune-related genes and immune checkpoints, and ER+ patients had better responses to immune checkpoint blocking. Correlation pathway research revealed that PPARG is strongly associated with pathways, such as angiogenesis, apoptosis, fatty acid biosynthesis, and degradation in ER+ BC. We also found that quercetin is the most promising natural anti-BC drug among natural medicines that upregulate PPARG. Our research showed that PPARG may reduce BC development by regulating the immune microenvironment. Quercetin as PPARG ligands/agonists is a potential natural drug for BC treatment.

## 1. Introduction

Breast cancer (BC) currently ranks first in incidence and second in mortality among cancers in females worldwide [1], representing a major health burden globally. Treatment methods for BC include surgery, radiotherapy, chemotherapy, endocrine therapy, and gene-targeted therapy, which depend on the underlying subtype and stage of BC. Despite significant progress in the field, the pathogenesis of BC remains unclear. Estrogen receptor positive (ER+) patients account for a higher proportion of all BC patients. The growth of ER+ tumor is driven by ER signal. Endocrine therapy is the main treatment. Representative drugs, such as tamoxifen, combined with radiotherapy and targeted therapy can effectively enhance the survival quality and prognosis of patients. Therefore, ER+ patients tend to have a better prognosis than estrogen receptor-negative (ER−) patients, but resistance inevitably develops over time, and drug resistance will gradually emerge. The therapeutic effect of second-line drugs is generally weaker than that of first-line drugs. Despite significant advances in diagnosis and treatment, some BC patients still have poor outcomes and prognosis. Finding new therapeutic targets and prognostic markers for BC is important to improve the efficiency and accuracy of BC treatment.

Peroxisome proliferator-activated receptor (PPAR) is a type of ligand-activated transcription factor that belongs to the nuclear receptor superfamily [2]. It participates in the control of lipid and carbohydrate turnover and their homeostasis and has important roles in cell differentiation and apoptosis, inflammation, vascular biology, and cancer [3]. Peroxisome proliferator-activated receptor gamma (PPARG) is the focus of research and a key factor in the regulation of lipid metabolism and energy homeostasis. It is an important treatment target for various metabolic diseases, inflammatory responses, cardiovascular diseases, and a variety of tumors [4, 5]. PPARG is also a key factor in immune regulation, since it has the ability to directly bind to DNA and activate transcription of target genes in immune cells [6–8]. PPARG is an important promoter of macrophage differentiation and M2 macrophage polarization [9, 10] and controls the lipid metabolism of various immune cells [9, 11–13]. The lipid microenvironment is associated with immune cell function in combination with classical transactivation. In an inflammatory response, PPARG can competitively inhibit the transduction of NF-*κ*B, JAK-STAT, and other signaling pathways, and inhibit the transactivation activity of pro-inflammatory transcription factors induced by cytokines, regulating the function and activity of macrophages, B cells, T cells, DC cells, and other immune cells. Its ligand reduces the damage caused by inflammatory responses to the body by inhibiting macrophage activation and inflammatory cytokine production. For example, the combination of anti-inflammatory drugs for experimental inflammatory bowel disease (IBD) and PPARG may become a new method for the treatment of IBD [14]. However, the current study on whether overexpression of PPARG affects the immune microenvironment of BC is not sufficient, and the mechanism is not well understood. Using bioinformatics, we studied PPARG expression and prognostic value in BC, its effect on immune cell infiltration, and immune checkpoints to better investigate the biological role of PPARG in BC cells. Further research is needed to explore potential natural drugs targeting PPARG in the treatment of BC, providing new insights into the detection and treatment of BC.

## 2. Methods

### 2.1. Pan-Cancer PPARG Expression Analysis

We obtained tumor data and associated clinical information from the Cancer Genome Atlas (TCGA; https://portal.gdc.cancer.gov/) and Genotype-Tissue Expression (GTEx; https://www.gtexportal.org/) databases. In addition, we employed the Wilcoxon test to examine the differential expression of PPARG in cancer and normal tissues. Statistical analysis was performed using version 4.0.3 of the R software. To be considered statistically significant, the criterion for *p*-value was set at less than 0.05.

### 2.2. Association Analysis between PPARG Expression and Clinical Characteristics of BC

We retrieved BC RNAseq data along with relevant clinical information from the TCGA database. The BC samples were categorized into high and low expression groups based on the median level of PPARG gene expression. Clinicopathological characteristics were analyzed in relation to PPARG gene expression. Variables studied included survival status, age, gender, pathologic tumor stage (pT-stage), pathologic node stage (pN-stage), pathological metastasis stage (pM-stage), and pathological tumor-node-metastasis stage (pTNM-stage). The data were expressed as mean ± SD, and unpaired *t*-tests were used for statistical evaluation. The association between PPARG and clinical characteristic variables was investigated using chi-square or Fisher's exact tests.

### 2.3. Prognostic Value Analysis of PPARG Gene in BC

We utilized BC RNAseq data and corresponding clinical information acquired from TCGA. The survival curve was generated using the “survminer” and the “Survival” software packages in R v4.0.3 to study the relationship between PPARG expression level and BC prognosis. Statistical analysis was performed using log-rank testing and univariate Cox regression to derive the *p*-values, hazard ratios (HR), and 95% confidence intervals (CI). A *p*-value lower than 0.05 was used to define statistical significance. Subsequently, we further investigated the prognostic value of the PPARG gene in BC by utilizing the Kaplan–Meier plotter (https://kmplot.com/analysis/).

### 2.4. Analysis of the Correlation between PPARG and Immune Infiltration in BC

We first utilized Tumor Immune Estimation Resource (TIMER) (https://cistrome.shinyapps.io/timer/) to reveal the correlation between PPARG and the infiltrating levels of six different immune cell subtypes, as well as the relationship between immune cell infiltration levels and BC patients' cumulative survival rate. Then, we obtained RNAseq data and corresponding clinical information of estrogen receptor-positive BC from TCGA database, and verified the relationship among PPARG and six immune cell subtypes infiltration levels using Spearman's correlation analysis. The correlation plot was implemented using the R v4.0.3 software package “ggstatsplot”, and A *p*-value below 0.05 indicates statistical significance.

### 2.5. Co-Expression Analysis of PPARG and Immune-Related Genes

Using BC RNAseq data and related clinical information from the TCGA database, the correlation between two genes was analyzed using “ggstatsplot” package in the R software with Spearman's correlation analysis for non-normally distributed quantitative variables. Additionally, the expression differences of immune checkpoint-related genes between ER+ and ER− BC were analyzed using “ggplot2” and “pheatmap” packages in the R software. Ultimately, the tumor immune dysfunction and exclusion (TIDE) algorithm was utilized to predict potential efficacy of immunotherapy [15]. Statistical significance is demonstrated when the *p*-value is equal to or less than 0.05.

### 2.6. Analysis of the Correlation between PPARG and Pathways

We utilized BC RNAseq data obtained from the TCGA database and corresponding clinical information. Gene sets containing relevant pathways were collected [16] and analyzed using the gene set variation analysis package in the R software version 4.0.3 with the parameter method = “ssgsea.” Finally, we used the Spearman's correlation analysis method to investigate the correlation between PPARG gene and pathway scores. A *p*-value below 0.05 was deemed to be statistically significant.

### 2.7. Mining of Potential Natural Compounds Regulating PPARG for BC Treatment

The BenCaoZuJian (HERB) database, a specialized high-throughput experimental and reference database for traditional Chinese medicine, was used to search for active compounds and herbal medicines targeting the PPARG receptor. Relevant data were extracted using reference mining, and searched the PubMed database to identify experimentally validated active compounds and natural drugs that regulate PPARG.

## 3. Results

### 3.1. Analysis of PPARG Expression in Pan-Cancer and ER+ BC

**Figure 1:**
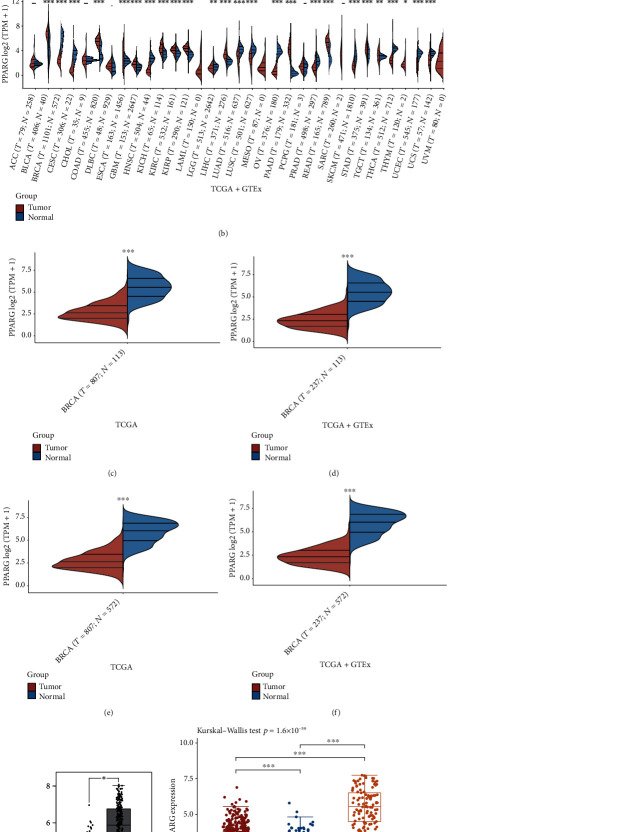
Pan-cancer and ER+/− BC analysis of PPARG expression. (a and b) PPARG expression in tumor and normal tissues in TCGA and TCGA + GTEx pancarcinoma data, the result shows that PPARG is downregulated in BC. (c and d) PPARG expression in ER+ BC and normal tissues in TCGA and TCGA + GTEx data, compared with normal tissues, PPARG is downregulated in ER+ BC. (e and f) PPARG expression in ER− BC and normal tissues in TCGA and TCGA + GTEx data, compared with normal tissues, PPARG is downregulated in ER− BC. (g) PPARG expression in BC and normal tissues in GEPIA data, the expression of PPARG is lower in BC than in normal tissue. (h) Differential expression of PPARG in ER+ and ER− BCs, the expression of PPARG is higher in ER+ BC than in ER− BC. ∗*p* < 0.05, ∗∗*p* < 0.001, and ∗∗∗*p* < 0.0001.

To research PPARG expression in pan-cancer and BC, we obtained RNAseq data and corresponding clinical information from 33 cancer types and 10,228 samples from TCGA and GTEx databases. First, we evaluated the PPARG expression in pan-cancer data from TCGA and GTEx. Results showed that PPARG was lowly expressed in 12 cancer types, including BC (BRCA), CESC, COAD, HNSC, LUAD, LUSC, OV, SKCM, PRAD, THCA, UCEC, UCS, KIRP, LIHC, STAD, KICH, KIRC, PAAD, and READ (see Figures 1(a) and 1(b)). Next, we evaluated the expression of PPARG in ER+, ER− BC, and normal tissue. We found that PPARG was lowly expressed in both ER+ and ER− BC compared with normal tissue (see Figures 1(c), 1(d), 1(e), and 1(f)). Furthermore, we further validated the low expression of PPARG in BC tissues using the Gene Expression Profile Interaction Analysis (GEPIA) online tool (http://gepia.cancer-pku.cn/; see Figure 1(g)). Furthermore, we analyzed the relationship between PPARG levels and ER status in BC and found that PPARG expression was higher in ER+ BC than in ER− BC (see Figure 1(h)). Taken together, these results suggest that PPARG is lowly expressed in BC.

### 3.2. PPARG Expression Levels in BC Patients in relation to Clinicopathological Characteristics

**Figure 2:**
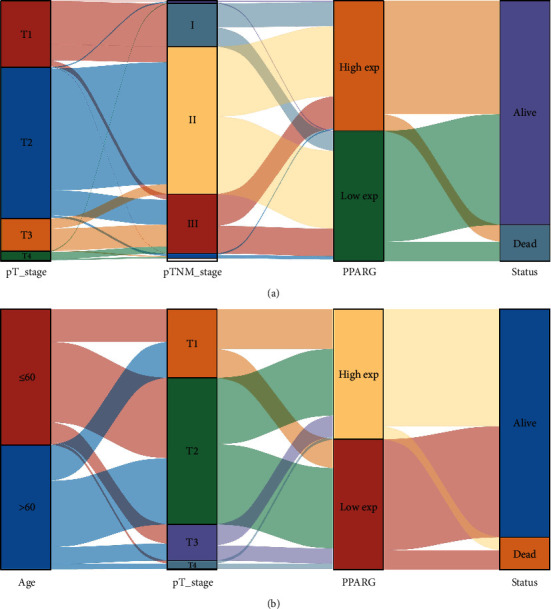
PPARG expression levels in BC patients in relation to clinicopathological characteristics. Each column represents a feature variable, with varying colors denoting different subtypes or stages, and the lines depicting the distribution of the same sample across the distinct feature variables. (a) PPARG expression level was related to pT-stage and pTNM-stage BC. (b) PPARG expression levels correlated with survival status, age, and pT-stage of ER+ BC.

We obtained RNAseq data and associated clinical information of 1101 BC cases from the TCGA database. The cases were categorized into high-expression and low-expression groups according to the median level of PPARG gene expression. We examined the correlation between PPARG expression and clinicopathological features. The outcome indicated that PPARG expression level was related to pT-stage and pTNM-stage of BC (see Table 1 and Figure 2(a)). In ER+ BC, PPARG expression levels correlated with survival status, age, pT-stage, and pTNM-stage (see Table 2 and Figure 2(b)). This result suggests that PPARG may be implicated in the pathogenesis of BC, particularly in ER+ BC, and may hold promise as a prognostic indicator.

### 3.3. Prognostic Value of PPARG in BC

**Figure 3:**
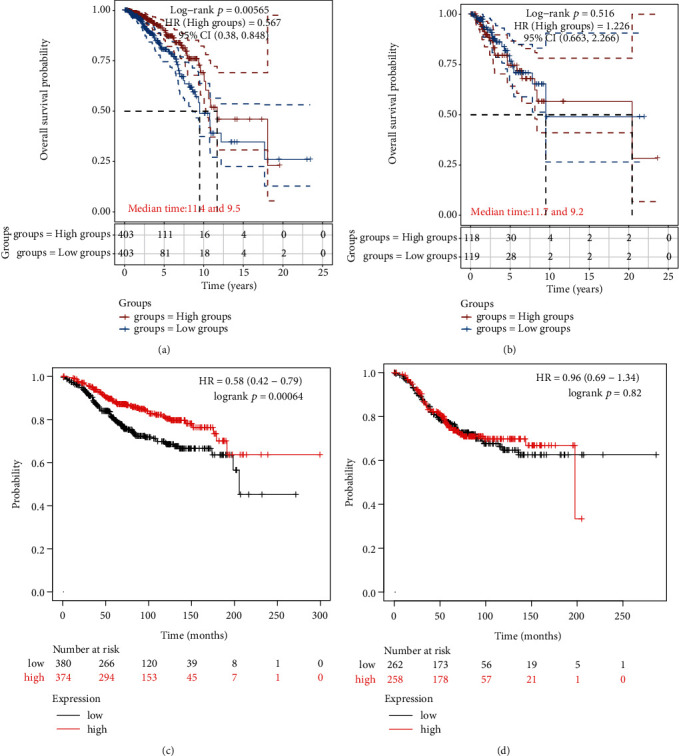
PPARG expression and the prognosis of BC. The KM survival curve of the PPARG gene in TCGA data is shown, wherein diverse groups were analyzed utilizing the log-rank test. HR (High exp.) represents the HR between the high and low expression groups. An HR > 1 indicates that the gene is a risk factor (higher expression is associated with poorer prognosis), whereas an HR < 1 indicates that the gene is a protective factor (higher expression is associated with better prognosis). The 95% CI represents the range of HR values with a certain level of certainty. Median time represents the time at which the survival rates of the high expression and low expression groups intersect at 50% (i.e., the median survival time). (a) Kaplan–Meier analysis of overall survival for ER+ BC in TCGA, PPARG gene is a protective factor in ER+ BC. (b) Kaplan–Meier analysis of overall survival for ER− BC in TCGA, survival of patients with ER− BC is not associated with the expression level of PPARG. (c) Kaplan–Meier analysis of overall survival for ER+ BC in Kaplan–Meier plotter, PPARG gene is a protective factor in ER+ BC. (d) Kaplan–Meier analysis of overall survival for ER− BC in in Kaplan–Meier plotter, survival of patients with ER− BC is not associated with the expression level of PPARG.

To evaluate the value of PPARG in predicting the prognosis of BC patients, we obtained RNAseq data and relevant clinical information from the TCGA database for 807 ER+ BC patients and 237 ER− BC patients. We applied survival correlation analysis to research the correlation among PPARG expression and BC prognosis. The results of the KM survival analysis showed that PPARG was a protective factor in ER+ BC (*p* = 0.0057), with higher expression associated with better prognosis (see Figure 3(a)). The corresponding survival times at 50% for the high expression and low expression groups were 11.4 and 9.5 years, respectively. However, there was no correlation between PPARG expression level and survival in ER− BC patients (see Figure 3(b)). We further validated these results using the online Kaplan–Meier plotter (http://kmplot.com/analysis/; see Figures 3(c) and 3(d)). Overall, these findings highlight the potential of PPARG as a therapeutic target and prognostic biomarker for ER+ BC.

### 3.4. PPARG Expression Is Associated with BC Immune Microenvironment

**Figure 4:**
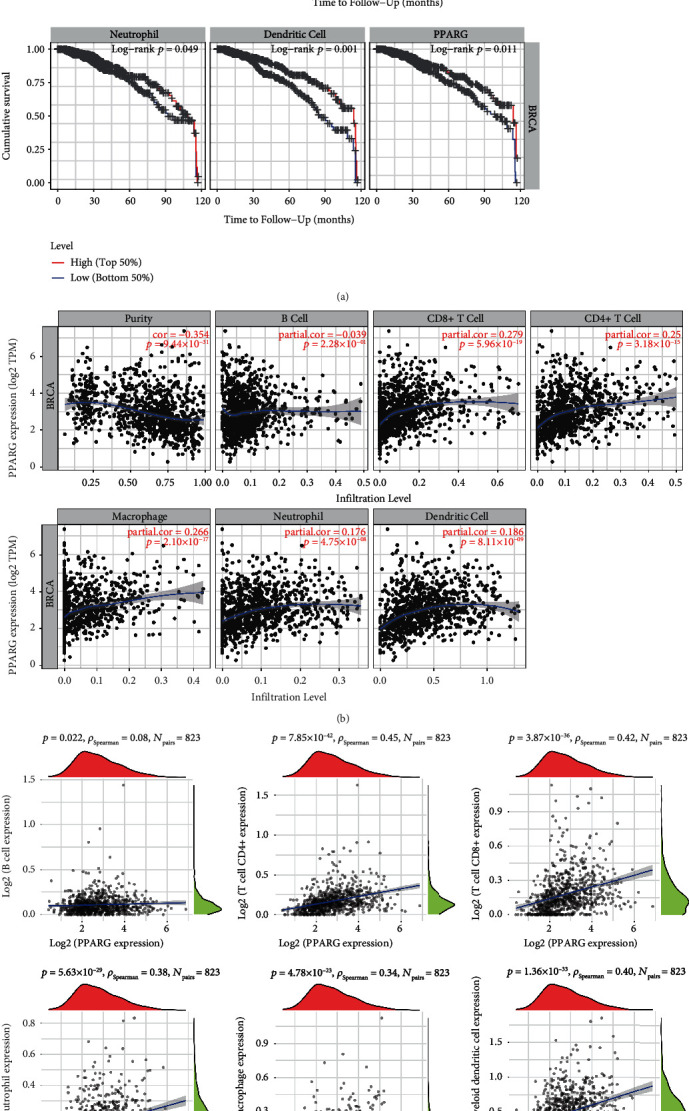
PPARG expression correlates with the immune microenvironment of BC. (a) Correlation between immune cell infiltration level and BC cumulative survival rate. (b) Correlation between PPARG expression levels and BC immune cell infiltration degree. (c) Correlation between PPARG expression and immune score in ER+ BC. The horizontal axis in the figure represents the distribution of the expression level of the first gene, whereas the vertical axis represents the distribution of the immune score. The right density curve shows the trend of immune score distribution, whereas the top density curve shows the trend of gene distribution. The correlation *p*-value and coefficient, as well as the method used to calculate the correlation, are indicated at the top.

To investigate the mechanisms behind the better prognosis associated with high PPARG expression, we utilized the TIMER tool to discover a link between PPARG and the degree of infiltration of six immune cell subtypes. The results showed that BC patients with higher levels of immune cell infiltration had better cumulative survival rates compared with those with lower levels of infiltration (see Figure 4(a)). Additionally, PPARG expression was shown to be positively related to the level of infiltration of CD8+ T cells (Cor = 0.279, *p* = 5.96 × 10^−19^), CD4^+^ T cells (Cor = 0.25, *p* = 3.18 × 10^−15^), macrophages (Cor = 0.266, *p* = 2.1 × 10^−17^), neutrophils (Cor = 0.176, *p* = 4.75 × 10^−8^), and dendritic cells (Cor =0.186, *p* = 8.11 × 10^−9^), with CD8^+^ T cells having the highest correlation (see Figure 4(b)). Based on the presented data, it can be concluded that patients with high expression of PPARG in BC exhibit better cumulative survival rates. This finding is corroborated by the results displayed in Figure 3. Furthermore, we obtained RNAseq data and related clinical data of ER+ BC from the TCGA database, and Spearman's correlation analysis confirmed the relationship between PPARG and the degree of infiltration of six immune cell subtypes (see Figure 4(c)). The results indicate that high PPARG expression is intimately linked to the immunological microenvironment of BC. This suggests that PPARG potentially exerts a crucial function in regulating the immune microenvironment of BC, which could have significant clinical implications for the development of novel therapeutic techniques for BC therapy.

### 3.5. Gene Co-Expression Analysis

**Figure 5:**
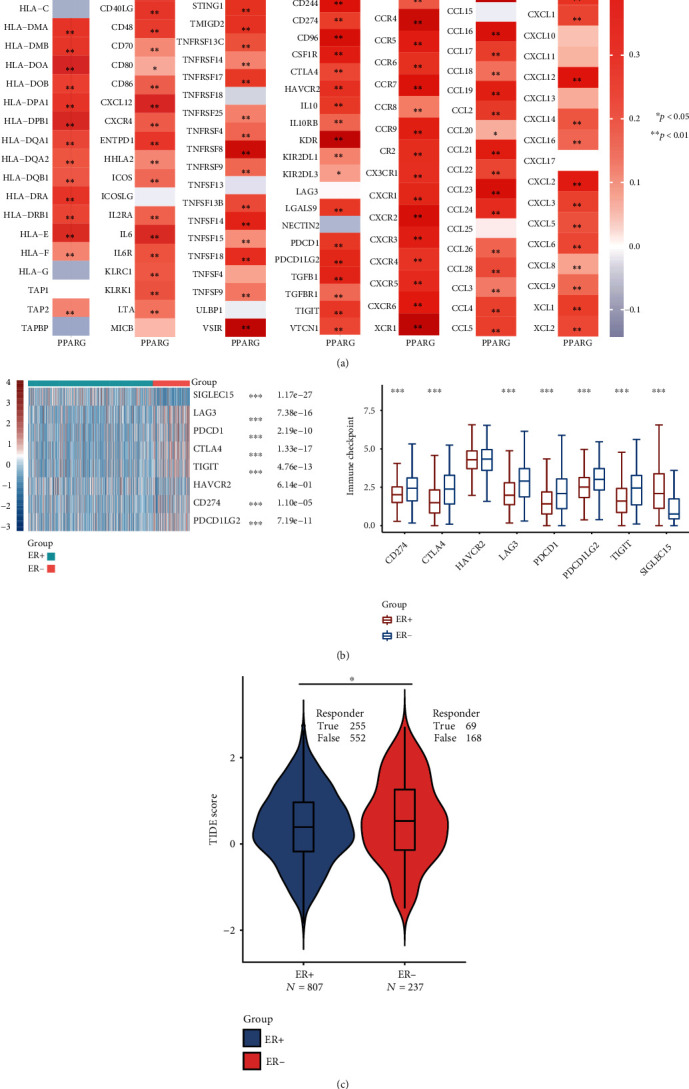
Co-expression analysis of PPARG, immune-related genes, and immune checkpoint molecules in BC. (a) Co-expression of PPARG with MHC genes, immunoactivation genes, immunosuppressive genes, chemokine receptor-related genes, and chemokine-related genes. (b) Different expression of immune checkpoints between ER+ and ER− BCs, ∗∗∗*p* < 0.001. (c) Different responses of ER+ and ER− BCs to immune checkpoint blocking therapy, ∗*p* < 0.05.

To evaluate the mechanism by which PPARG is associated with immune cells in ER+ BC, we performed gene co-expression analysis. MHC genes, immune activation genes, immunosuppressive genes, and chemokine (receptor) related genes were studied. PPARG is co-expressed with all chemokine receptors listed, and its expression level is positively correlated with most chemokines. Meanwhile, PPARG expression was shown to be positively linked with the majority of MHC genes, such as HLA-DOA, HLA-DPB1, HLA-DRA, and HLA-E genes. It is noteworthy that the expression of PPARG is positively correlated with almost all immune suppressor genes (see Figure 5(a)).

We further compared the expression of immune checkpoints, which are molecules expressed on immune cells that inhibit immune cell function, leading to ineffective anti-tumor immune responses and tumor immune evasion, between ER+ and ER− BC. The results showed that immune checkpoints SIGLEC15 (*p* = 1.17 × 10^−27^), LAG3 (*p* = 7.38 × 10^−16^), PDCD1 (*p* = 2.19 × 10^−10^), CTLA4 (*p* = 1.33 × 10^−17^), TIGIT (*p* = 4.76 × 10^−13^), CD274 (*p* = 1.10 × 10^−5^), and PDCD1LG2 (*p* = 7.19 × 10^−11^) were expressed at lower levels in ER+ BC than in ER− BC (see Figure 5(b)). We found that ER+ patients exhibit stronger responses to immune checkpoint blockade (ICB) compared with ER− patients (see Figure 5(c)). A higher TIDE score is associated with reduced effectiveness of ICB therapy and shorter survival following such treatment [16]. Furthermore, the results showed that PPARG was co-expressed with these immune checkpoints (Table 3), indicating the potential of PPARG as an immunotherapy target.

### 3.6. Correlation Analysis between PPARG and Pathway

**Figure 6:**
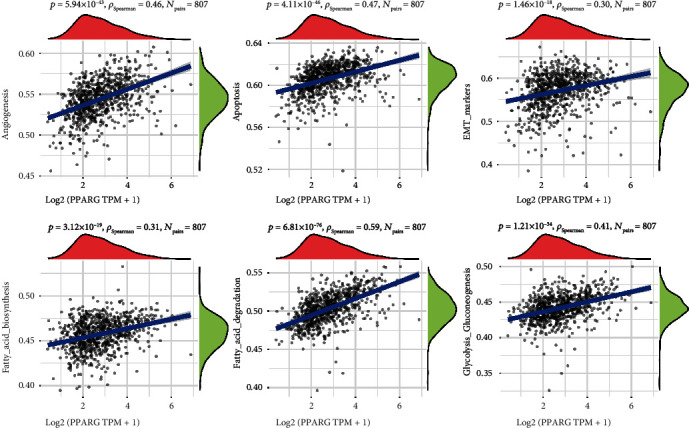
Spearman's correlation analysis between PPARG and path score. The*x*-axis in the picture shows gene expression, whereas the *y*-axis reflects pathway score. The right density curve depicts the trend of the pathway score distribution, whereas the topmost curve portrays the trend of distribution in gene expression level. The top numerical value (shown by the blue curve within the coordinate axis) indicates the correlation *p*-value, correlation coefficient, and method used to calculate the correlation between a single gene and pathway score.

We obtained RNAseq data and associated clinical information for ER+ BC from TCGA database. The statistical analysis revealed that PPARG is closely associated with various pathways, including angiogenesis, apoptosis, epithelial–mesenchymal transition (EMT) markers, fatty acid biosynthesis, fatty acid degradation, and glycolysis–gluconeogenesis, in estrogen receptor-positive BC (see Figure 6). Given its involvement in several critical pathways that contribute significantly to tumor growth, progression, and metastasis, these findings provide additional evidence to support the potential targeting of PPARG for the treatment of ER+ BC. Therefore, by modulating the expression or activity of PPARG, it may be possible to interfere with these pathways and inhibit tumor growth and metastasis. These discoveries offer a foundation for the development of novel PPARG-related BC treatment approaches.

### 3.7. Regulation of PPARG by Natural Drugs

**Figure 7:**
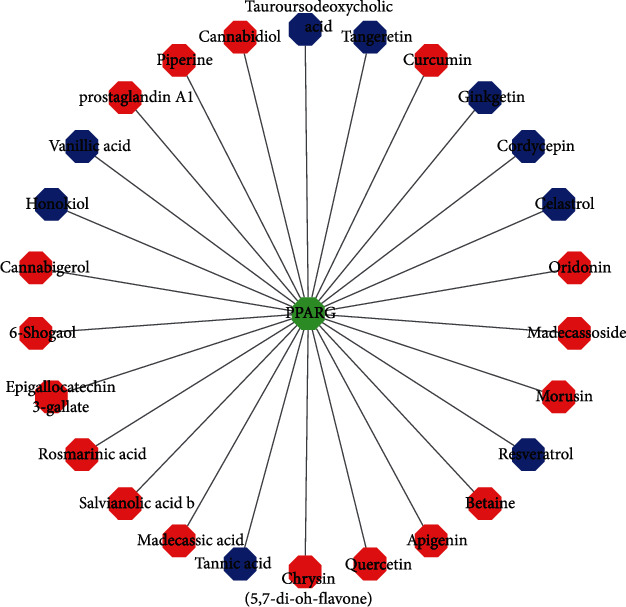
Regulation of PPARG by natural drugs (red represents up-regulated natural drugs, whereas blue represents down-regulated natural drugs).

We utilized the HERB database to search for active compounds and Chinese herbal medicines targeting the PPARG receptor, and identified experimentally verified active compounds and natural drugs. Natural drugs that up-regulate PPARG include apigenin [17], betaine [18], morusin [19], madecassoside [20], oridonin [21], curcumin [22], cannabidiol [23], piperine [24], prostaglandin A1 [25], 6-shogaol [26], epigallocatechin 3-gallate [27], rosmarinic acid [28], salvianolic acid b [29], madecassic acid [30], chrysin (5,7-di-OH-flavone) [31], and quercetin [32]. Natural drugs that down-regulated PPARG included resveratrol [33], celastrol [34], cordycepin [35], ginkgetin [36], tangeretin [37], tauroursodeoxycholic acid [38], vanillic acid [39], honokiol [40], and tannic acid [41] (see Figure 7). As discussed earlier, these results suggest that natural drugs that up-regulate PPARG may have therapeutic potential in treating ER+ BC, whereas those that down-regulate PPARG may have a negative impact on the treatment outcome. This provides a basis for the development of new natural drugs or drug combinations for further investigation of their potential in treating ER+ BC.

## 4. Discussion

ER+ BC is the most common subtype of BC. While endocrine therapy reduces BC recurrence and mortality, acquired resistance developed during treatment remains a significant challenge [42]. Drug resistance mechanisms involve various factors, such as the tumor immune microenvironment, gene regulation, estrogen and comodulated cofactors, growth factor signaling pathways, autophagy and apoptosis mechanisms, non-coding RNA regulation, and immune surveillance [43]. Currently, tumor immunity and immunotherapy have become the forefront of tumor research and are recognized as important anti-tumor pathways. The prognosis and treatment of BC are strongly associated with the stage and subtype of BC. Therefore, it is crucial to explore immune-related prognostic factors that are more generally applicable to immunotherapy of BC. These findings provide a basis for developing new natural drugs or drug combinations for further investigating their potential in the treatment of ER+ BC.

The tumor microenvironment (TME) is crucial in the progression of tumors [44], and the responsiveness of BC patients to immunotherapy depends on the dynamic response among tumor cells as well as immune infiltrating cells in TME. PPARG belongs to the ligand-activated transcription factor family and it is expressed in a variety of immune cells. It plays a critical role in various immunological processes, such as energy metabolism, cell division, inflammatory response, and cancer development and progression. Therefore, targeting PPARG may hold promise as an immunotherapy approach for BC and be associated with drug resistance and prognosis based on TME infiltration characterization of cancer tissue. Clinical studies have demonstrated the key role of PPARG in tumorigenesis and development in various types of tumors, including BC, liver cancer, lung cancer, and neurological tumors, through the inhibition of cancer cell proliferation or the promotion of cancer cell apoptosis and autophagy. However, our understanding of PPARG in BC remains incomplete, and there are few studies on its differential expression in different types of BC and its relevance with BC prognosis, which requires further in-depth study.

From this study, we first found that PPARG was poorly expressed in BC. (Figures 1(a) and 1(b)). We then analyzed different types of BC and found that PPARG was under-expressed in both ER+ and ER− BC (Figures 1(c), 1(d), 1(e), and 1(f)), whereas PPARG expression is higher in ER+ BC compared with ER− BC (Figure 1(h)). These results demonstrate that PPARG is expressed differently in different types of BC. Next, we evaluated the relationship between PPARG expression levels and clinicopathological variables from a clinical perspective. We discovered that the level of PPARG expression was associated with BC pT-stage and pTNM-stage (Table 1 and Figure 2(a)), and correlated with the survival status and pT-stage of ER+ BC (Table 2 and Figure 2(b)). To analyze the prognostic value of PPARG gene in BC, we used Kaplan–Meier and verified the previous results (Figure 3(c) and 3(d)). This is consistent with the findings of Jiang et al. [45]. With larger BC tumor size, the occurrence of axillary lymph node metastasis, and the increase of BC histological grade and TNM stage, PPARG expression level decreased significantly. High expression of PPARG often represents a higher overall survival rate.

There are many kinds of immune cells infiltration in TME. Studying the regulation of PPARG on immune cell infiltration levels in the TME is important to clarify its effects on BC development, metastasis, treatment, and drug resistance. PPARG not only regulates macrophage differentiation and polarization [46], but also regulates lipid metabolism of immune cells [47, 48], inhibits the production of various cytokines, such as TNF*α*, IL-1B, and IL-6 [49, 50], downregulates chemokines and receptors (IL-12, CD80, CXCL10, and RANTES), and recruits Th1 lymphocytes. PPARG can alter gene expression independently of DNA binding, and this type of transrepression may be the main molecular mechanism driving the function of macrophages, dendritic cells, and T cells in terms of their phenotype and secretory output [4], making PPARG associated with the dynamic regulation of TME. When exploring the correlation between PPARG expression and the immune microenvironment in BC, we selected the six cells mentioned above as study cells. We found that the cumulative survival rate of BC patients with high immune cell infiltration levels was better (see Figure 4(a)). Spearman's correlation analysis results also verified the correlation of PPARG with the level of infiltration of six immune cell subtypes (see Figure 4(c)), confirming that PPARG expression was positively correlated with these cells (see Figure 4(b)). The aforementioned findings indicate that BC patients with high expression of PPARG exhibit relatively better overall survival prognosis, which is consistent with the results depicted in Figure 3.

The results of our co-expression analysis showed that PPARG was positive for co-expression with all listed chemokine receptors and positively correlated with most MHC genes. We found that CCR7 and CXCR2 of neutrophils, as well as CSF1R and CCL16 of macrophages were significantly correlated with PPARG expression in BC. These results suggest that PPARG may regulate macrophage polarization in BC. The expression of dendritic cell markers HLA-DPB1, HLA-DRA, and HLA-DPA1 were significantly correlated with the expression of PPARG, suggesting a close relationship between PPARG expression and the infiltration level of dendritic cells. Since dendritic cells can promote tumor progression by cross-presenting tumor antigens to activate the cross-initiating process of CD8^+^ T cells [15], this finding is significant. Notably, almost all immunosuppressive genes were co-expressed with PPARG. The mechanism may be related to PPARG's regulation of the balance between immune cell infiltration and immunosuppression. On the one hand, it can enhance the chemotaxis and retention of immune cells and promote the beneficial immune response to kill tumor cells. On the other hand, the expression of immunosuppressive genes can be regulated by inhibiting the activity of immune cells to avoid the excessive immune response leading to normal tissue damage. In addition, PPARG may suppress the immune response by participating in the regulation of polarization of M2-type macrophages. More possible regulatory mechanisms need to be further explored.

Recent findings suggest that PPARG can affect a variety of biological functions by regulating and expressing different signaling pathways, such as *β*2-adrenaline promoting of BC growth and angiogenesis through the downregulation of PPARG [51], and as a PPAR*γ* agonist, VSP-17 is capable of inhibiting the process of EMT, thereby suppressing the migration and invasion of triple-negative BC cells, through the PPARG/AMPK signaling pathway [52]. Correlation analysis of PPARG with pathways reveals that PPARG is highly correlated with angiogenesis, apoptosis, EMT markers, fatty acid biosynthesis, fatty acid degradation, glycolysis–gluconeogenesis, and other pathways. These findings illustrate that PPARG might be a viable therapeutic target, BC patients with relatively high PPARG expression may have a better prognosis, and ligands/agonists of PPARG are a new way to treat advanced BC.

By searching the HERB database, we have discovered that some natural drugs are capable of regulating the expression of PPARG. Among these drugs, those that upregulate the expression of PPARG may have potential for use in treating and preventing BC, which could lead to improved prognosis and better outcomes for BC patients. Quercetin and curcumin are two natural drugs that have received a lot of attention due to their promising research findings. According to recent research, quercetin has been shown to increase adiponectin secretion and prevent atherosclerosis by regulating factors, such as PPARG [53]. Additionally, it has been demonstrated to inhibit the development and progression of BC and other tumors [54]. Specifically, quercetin has a potent anti-tumor effect by inducing reactive oxygen species (ROS)-dependent apoptosis in MCF-7 BC cells, and it also induces apoptosis in human BC cells by activating PTEN to inhibit the PI3K/AKT and JNK signaling pathways [55, 56]. Moreover, quercetin nanoparticles have been found to exhibit in vitro efficacy and in vivo safety, making them a promising potential anti-BC agent [57].

Curcumin interferes with the EMT process and inhibits BC cell migration, inducing BC apoptosis and cell death [58, 59]. Other natural drugs that upregulate PPARG include apigenin, betaine, morusin, madecassoside, oridonin, piperine, prostaglandin A1, cannabigerol, and others. Several flavonoids, such as apigenin, have been studied for the treatment of experimental colitis [14, 60], Apigenin inhibits p65 translocation to the nucleus by activating PPARG, reduces the expression of NF-*κ*B, and contributes to the polarization of M2 macrophages. It also alleviates hepatic and muscle steatosis [17]. Cannabinol can regulate human metabolism, reduce *β*-amyloid toxicity and inflammation in rats through PPARG antagonism, and induce apoptosis through PPARG, which has therapeutic effects on liver, cervical, and lung cancers [61]. These natural compounds and active ingredients have been shown to be novel PPARG ligands in clinical trials, and their therapeutic effects and clinical value for other diseases, including BC, warrant further exploration.

## 5. Conclusion

Our study concludes that downregulation of PPARG is linked with poor prognosis in BC. PPARG may regulate tumor-infiltrating cells in the TME through different pathways, thereby affecting tumor development. PPARG could be a promising target for BC treatment, and natural products and compounds from traditional Chinese medicine can modulate its expression, offering a new therapeutic approach for BC treatment.

## Figures and Tables

**Table 1:** PPARG expression levels in BC patients in relation to clinicopathological characteristics.

	Clinicopathological characteristics	High expression group	Low expression group	*p-*Value
Status	Alive	480	467	0.333
	Dead	71	83	
Age	Mean (SD)	57.6 (13)	59.1 (13.4)	0.069
	Median [Min, Max]	58 [26, 90]	59 [26, 90]	
Gender	Female	546	543	0.769
	Male	5	7	
pT-stage	TX	0	3	0.007
	T1	161	120	
	T2	292	347	
	T3	83	55	
	T4	15	25	
pN-stage	NX	7	13	0.173
	N0	252	264	
	N1	187	179	
	N2	56	64	
	N3	49	30	
pM-stage	MX	86	77	0.182
	M0	455	455	
	M1	7	15	
pTNM-stage	X	5	8	0.041
	I	102	80	
	II	296	328	
	III	136	115	
	IV	6	14	

**Table 2:** PPARG expression levels in ER+ and ER− BC patients in relation to clinicopathological characteristics.

		Clinicopathological characteristics	High expression group	Low expression group	*p-*Value
Status	ER+	Alive	364	343	0.041
		Dead	40	60	
	ER−	Alive	97	99	0.754
		Dead	22	19	
Age	ER+	Mean (SD)	58.2 (13)	60.5 (13.5)	0.015
		Median [Min, Max]	58 [26, 90]	61 [29, 90]	
	ER−	Mean (SD)	56.1 (11.8)	55.7 (12.9)	0.789
		Median [Min, Max]	55 [29, 85]	54.5 [26, 90]	
Gender	ER+	Female	399	396	0.768
		Male	5	7	
	ER−	Female	119	118	≤0.001
		Male	0	0	
pT-stage	ER+	TX	0	2	0.025
		T1	123	90	
		T2	207	247	
		T3	65	47	
		T4	9	17	
	ER−	TX	0	1	0.982
		T1	29	25	
		T2	72	77	
		T3	13	10	
		T4	5	5	
pN-stage	ER+	NX	6	10	0.182
		N0	181	175	
		N1	139	146	
		N2	39	51	
		N3	39	21	
	ER−	NX	1	1	0.375
		N0	61	75	
		N1	32	30	
		N2	15	7	
		N3	10	5	
pM-stage	ER+	MX	67	70	0.553
		M0	329	320	
		M1	6	10	
	ER−	MX	15	10	0.571
		M0	101	106	
		M1	2	2	
pTNM-stage	ER+	X	4	**7**	0.013
		I	81	60	
		II	211	231	
		III	100	92	
		IV	6	9	
	ER−	X	0	1	0.292
		I	19	16	
		II	65	82	
		III	31	15	
		IV	1	2	

**Table 3:** Correlation between PPARG and immune checkpoints in estrogen receptor-positive BC.

Genes	Cor	*p*-Value
CD274	0.235364	1.28 × 10^−11^∗∗
CTLA4	0.212676	1.05 × 10^−9^∗∗
HAVCR2	0.268371	8.86 × 10^−15^∗∗
LAG3	0.003241	0.926766
PDCD1	0.243537	2.32 × 10^−12^∗∗
PDCD1LG2	0.294025	1.48 × 10^−17^∗∗
TIGIT	0.248973	7.23 × 10^−13^∗∗
SIGLEC15	0.194715	2.46×10^−8^∗∗

∗∗*p* < 0.001.

## Data Availability

Data supporting this research article are available from the corresponding author or first author on reasonable request.
